# Sex-related variations in bone microstructure of rabbits intramuscularly exposed to patulin

**DOI:** 10.1186/s13028-015-0140-0

**Published:** 2015-09-03

**Authors:** Hana Duranova, Veronika Kovacova, Ramona Babosova, Radoslav Omelka, Maria Adamkovicova, Birgit Grosskopf, Marcela Capcarova, Monika Martiniakova

**Affiliations:** Department of Zoology and Anthropology, Constantine the Philosopher University, 949 74 Nitra, Slovakia; Department of Botany and Genetics, Constantine the Philosopher University, 949 74 Nitra, Slovakia; Institute of Zoology and Anthropology, Georg-August University, 37 073 Göttingen, Germany; Department of Animal Physiology, Slovak University of Agriculture, 949 76 Nitra, Slovakia

## Abstract

**Background:**

Patulin, a toxic mold metabolite, has been found as natural contaminant of processed fruits, most notably apples, apple juices and other apple-based products. A number of adverse health effects in humans and animals are associated with patulin intoxication. The current study was performed to analyse possible toxic effects of patulin on femoral bone microstructure in adult rabbits in detail. Fourteen clinically healthy four-month-old rabbits of both sexes (6 males and 8 females) were included in the study. Animals from the experimental groups (group E♂, n = 3; group E♀, n = 4) were injected intramuscularly with patulin at dose 10 μg/kg body weight two times a week for 28 days. The dose of patulin was estimated based on the maximum permitted level of patulin for apple products intended for infants and young children. Three males and four females without patulin administration served as controls (groups C♂ and C♀). Cortical bone thickness and qualitative and quantitative histological characteristics of compact bone tissue were investigated.

**Results:**

Intramuscular applications of patulin significantly increased the thickness of cortical bone in both sexes of rabbits. In patulin-exposed males, an absence of primary vascular longitudinal bone tissue near the endosteal border was observed, which could be associated with intensive bone remodeling. Femoral diaphyses of females displayed a lower number of secondary osteons in the middle part of the *substantia compacta*, and occurrence of the osteons near the periosteum. This could indicate alterations in bone turnover. Histomorphometrical evaluations showed significantly increased sizes of the primary osteons’ vascular canals (*P* < 0.05) in males exposed to patulin possibly due to mycotoxin-induced increased levels of testosterone.

**Conclusions:**

This study demonstrates significant impact of intramuscular application of patulin on bone microstructure in adult rabbits. Moreover, we have found that the effects of patulin on qualitative and quantitative histological characteristics of compact bone are sex-dependent.

## Background

Mycotoxins are secondary fungal metabolites produced by species of filamentous fungi growing on cereals and other food commodities under special conditions, e.g., high levels of humidity and temperature, before and/or during harvest, handling, shipping and storage [1, 2]. According to Hussein and Brasel [3], the worldwide mycotoxin contamination of foods and feeds represents a significant problem because mycotoxins are capable of causing disease and death of humans and animals [4]. Although hundreds of fungal toxins are known, patulin is one of the limited number generally considered to play important roles in food safety [5].

Patulin, 4-hydroxy-4H-furo[3,2-c]pyran-2(6H)-one, is a mycotoxin produced by several species of *Penicillium*, *Aspergillus* and *Byssochlamys* [6, 7]. This mycotoxin is mainly found in apples and apple products [6, 8, 9], and occasionally in pears, grapes, apricots, strawberries, blueberries and peaches [10]. It has been suggested that the presence of patulin may be indicative of the fruit quality used in production [11]. Based on valid international standards, a maximum permitted level of patulin is 50 μg/l in fruit products [12]. Recently, Food Additives (JECFA) has established a provisional maximum tolerable daily intake for patulin of 0.4 μg/kg body weight (bw)/day [13].

Despite its antibiotic properties, numerous adverse health impacts have been attributed to patulin [14]. The mycotoxin is believed to exert its cytotoxic [15, 16], genotoxic [16, 17], immunotoxic [18], and carcinogenic [19] effects mainly by forming covalent adducts with essential cellular thiols in proteins, as well as by causing glutathione depletion leading to oxidative damage and generation of oxidative stress [20, 21]. Puel et al. [22] state that affinity of patulin to sulfhydryl groups may explain its inhibitory effect on many enzymes. Moreover, patulin treatment was found to activate protein degradation, especially proteasome activities, and sulfur amino acid metabolism [23].

Toxicological studies have shown that patulin induces nephropathy and gastrointestinal tract malfunction in humans and animal models [17, 20, 21, 24]. Also, histopathological changes in reproductive organs (e.g., epididymis, prostate) [25] and the thymus [26] were observed in growing male rats exposed to patulin. According to Huff et al. [27] and Maurice et al. [28], mycotoxin-contaminated diets can affect growth and cause bone fragility and decreased bone strength. However, the effect of patulin on bone microstructure had not been investigated prior to our experiment.

The aim of the present study was to evaluate the possible toxic impact of patulin on bone microstructure in adult rabbits of both sexes. Additionally, sex-based differences in the osteotoxic action of patulin were assessed.

## Methods

### Animals

Adult male (n = 6) and female (n = 8) clinically healthy rabbits of meat line M91 (Californian broiler line), obtained from an experimental farm of the Animal Production Research Centre in Nitra, Slovak Republic, were used. In approximately 35 % of musculoskeletal research studies, rabbits are the first choice of animal mainly due to their reasonable anatomical size and lower costs in terms of purchase and maintenance as compared to larger animals [29]. An additional advantage is the fact that they reach skeletal maturity shortly after sexual maturity, around 5–6 month of age [30, 31]. Our rabbits (at the age of 4 months, weighing 3.5–4.0 kg) were housed in individual flat-deck wire cages (area 0.3 m^2^) under standard conditions (temperature 20–22 °C, humidity 55 ± 10 %, 12/12 h cycle of light and darkness) with access to food (feed mixture) and drinking water ad libitum. To investigate skeletal sexual dimorphism in toxic action of patulin, rabbits of both sexes were used.

The rabbits were randomly divided into four groups (E♂, E♀, C♂, and C♀). In the groups E♂ (n = 3) and E♀ (n = 4), adult rabbits were intramuscularly injected with patulin (Sigma Aldrich, Munich, Germany) at dose 10 μg/kg bw dissolved in saline two times per week for 28 days. The dose of patulin was chosen on the basis of experimental studies and previous experiments. It reflects the permitted limit of patulin in apples and apple products for infants and young children (non-toxic dose) [32]. The duration of the experiment (28 days) was set based on the period of an early stage of bone healing in rabbits (around 4 weeks).

To ensure that all rabbits received a complete dose, patulin was injected into the *musculus quadriceps femori*. This muscle was chosen as the injection site because of its considerable vascularization compared to other muscle groups. For the safety of our laboratory technician, the toxin was injected in a liquid form. The groups C♂ (n = 3) and C♀ (n = 4) served as controls and were injected intramuscularly with physiological saline solution at the same times. The rabbits were kept for other investigations (e.g., histological and biochemical analyses) at the Animal Production Research Centre in Nitra (Slovak Republic). The present study was performed as an additional investigation focused on bone microstructure. All procedures were approved by the Animal Experimental Committee of the Slovak Republic.

### Procedures

The rabbits were euthanized by electrocution 28 days after first exposure to patulin and their femurs were sampled for histological analyses. After all soft tissue had been removed, the right femurs were sectioned at the midshaft of the diaphysis and fixed in HistoChoice fixative (Amresco Inc., Solon, USA). The specimens were then dehydrated in increasing grades (40–100 %) of ethanol and embedded in Biodur epoxy resin (Günter von Hagens, Heidelberg, Germany) according to the method described by Martiniaková et al. [33]. Transverse 70–80 μm sections were prepared with a sawing microtome (Leitz 1600, Leica, Wetzlar, Germany) and fixed onto glass slides by Eukitt (Merck, Darmstadt, Germany) as previously described [34]. The qualitative histological characteristics of the compact bone were determined according to the internationally accepted classification systems of Enlow and Brown [35] and Ricqlés et al. [36], who classified bone tissue into three main categories: primary vascular tissue, non-vascular tissue and Haversian bone tissue. Various patterns of vascularization can occur in primary vascular bone tissue: longitudinal, radial, reticular, plexiform, laminar, lepidosteoid, acellular, fibriform and protohaversian. There are three subcategories identified in Haversian bone tissue: irregular, endosteal and dense. The quantitative (histomorphometrical) variables were assessed using the software Motic Images Plus 2.0 ML (Motic China Group Co., Ltd.). We measured area, perimeter and the minimum and maximum diameters of primary osteons’ vascular canals, Haversian canals and secondary osteons in all views (i.e., anterior, posterior, medial and lateral) in order to minimize differences in bone locations. Secondary osteons were distinguished from primary osteons (i.e., primary vascular canals) on the basis of the well-defined peripheral boundary (cement line) between the osteon and the surrounding tissue. Diaphyseal cortical bone thickness was measured by Motic Images Plus 2.0 ML software. Twenty random areas were selected, and average thickness was calculated for each femur.

### Statistics

Statistical analysis was performed using SPSS 8.0 software. All data were expressed as mean ± standard deviation (SD). The unpaired Student’s *t* test was used for statistical significance (*P* < 0.05).

## Results

No clinical signs or behavioral alterations were observed in rabbits in any of the groups throughout the course of the experiment.

### Qualitative histological characteristics

**Fig. 1:**
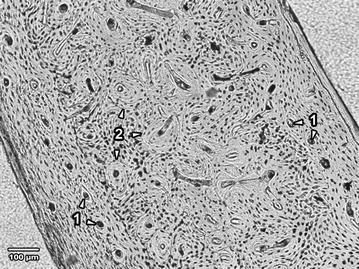
Microscopical structure of compact bone in rabbits from the control groups. *1* Primary vascular longitudinal bone tissue near endosteal and periosteal surfaces (*arrows* vascular canals of primary osteons). *2* Dense Haversian bone tissue creating the middle part of the *substantia compacta* (*arrows* secondary osteons)

The femoral diaphyses in male and female rabbits from the control groups displayed similar histological structure. Primary vascular longitudinal bone tissue, as a basic structural pattern of all bones, formed the inner layer surrounding the medullary cavity (endosteal border) and also periosteal surfaces of the *substantia compacta*. The tissue contained vascular canals which ran in a direction essentially parallel to the long axis of the bone. Near endosteal surfaces, primary vascular radial bone tissue composed of branching or non-branching vascular canals radiating from the marrow cavity, irregular and dense Haversian bone characterized by the occurrence of scattered secondary osteons and large numbers of secondary osteons, respectively, were also present. The middle part of the compact bone consisted of a layer of dense Haversian bone (Fig. 1).

Intramuscular administration of patulin caused marked changes in bone microstructure in rabbits of both experimental groups (E♂; E♀). Moreover, marked differences between sexes were found.

**Fig. 2:**
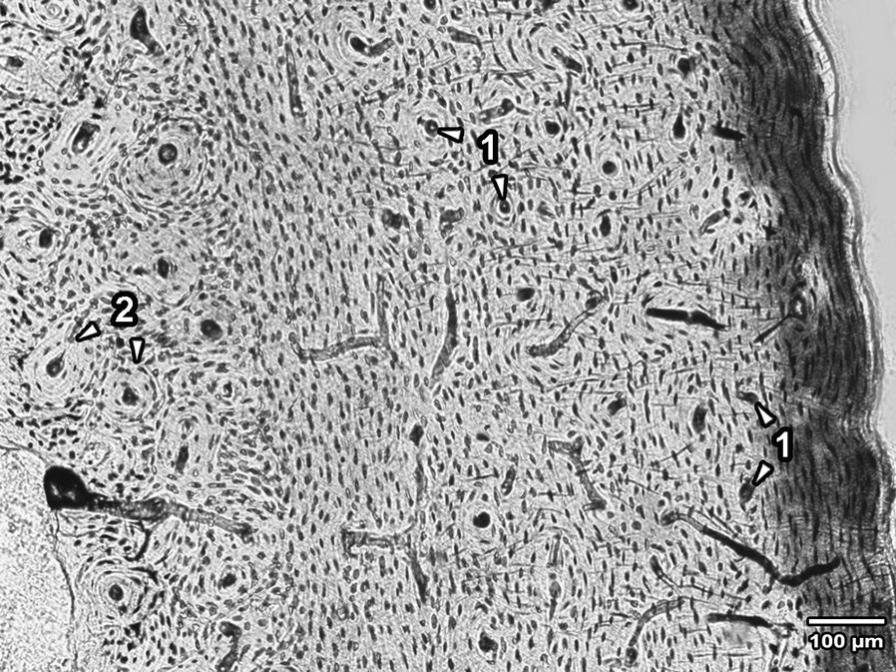
Microscopical structure of compact bone in rabbits from the group E♂. *1* Primary vascular longitudinal bone near periosteal surface and in the middle segment of the *substantia compacta* (*arrows* vascular canals of primary osteons with significantly decreased size). *2* Dense subendosteal Haversian bone (*arrows* secondary osteons)

**Fig. 3:**
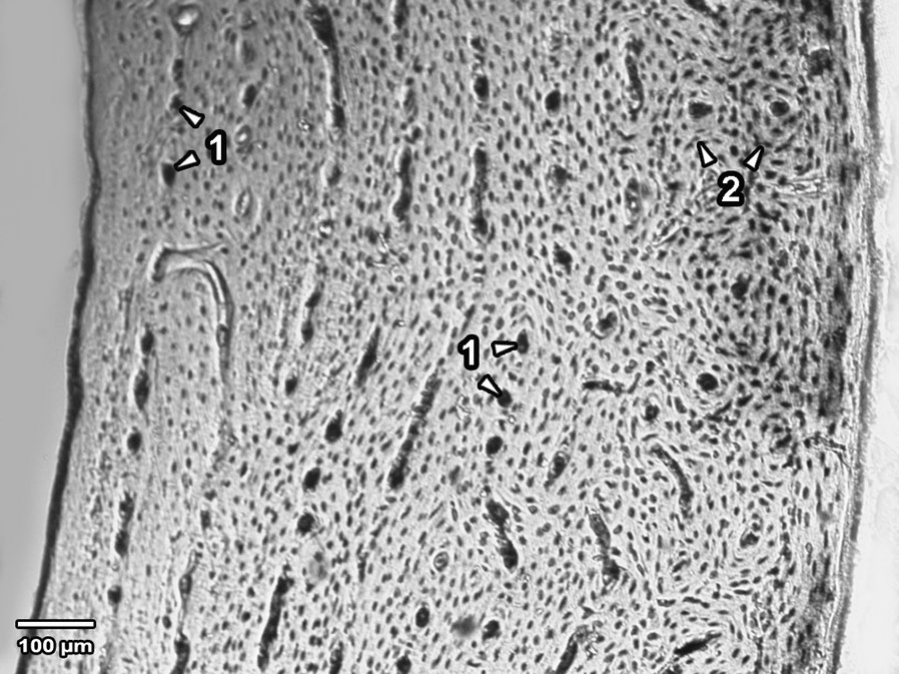
Microscopical structure of compact bone in rabbits from the group E♀. *1* Endosteal surface and the middle area of *substantia compacta* consisting of primary vascular longitudinal bone (*arrows* vascular canals of primary osteons). *2* Secondary osteons near the periosteum (*arrows* secondary osteons)

In the femoral diaphysis of male rabbits exposed to patulin, subendosteal primary vascular longitudinal bone tissue was absent. This part of the bone consisted only of dense Haversian bone tissue. The periosteal surface was formed by primary vascular longitudinal bone which often also created the middle part of the compacta (Fig. 2). In female rabbits from group E♀ (Fig. 3), a lower number of secondary osteons was observed in the middle part of the bone (*P* < 0.05). From this part of the bone, secondary osteons were marginalized to periosteum suggesting alterations in bone turnover. In some individuals, the middle part of the *substantia compacta* consisted of primary vascular longitudinal bone, which in group C♀ rabbits created endosteal and periosteal surfaces.

### Quantitative histological characteristics

The thickness of cortical bone were significantly higher (*P* < 0.05) in rabbits of both sexes exposed to patulin than in rabbits of the control groups (Table 1).

**Table 1:** Cortical bone thickness in adult male and female rabbits intramuscularly exposed to patulin at dose 10 μg/kg body weight two times per week for 28 days (E♂, E♀) and control rabbits (C♂, C♀)

Group of rabbits	N	N1	Cortical bone thickness (mm)	*P* value
C♂	3	72	997.51 ± 92.95	<0.05
E♂	3	72	1059.31 ± 118.93	
C♀	4	96	996.65 ± 96.28	<0.05
E♀	4	96	1068.18 ± 153.08	

*N* number of rabbits, *N1* number of measured structures

For the quantitative histological characteristics, totals of 575 vascular canals of primary osteons, 402 Haversian canals and 402 secondary osteons were measured. Data on bone measurements are shown in Tables 2 and 3. The values for all parameters of the primary osteons’ vascular canals were significantly lower in patulin-intoxicated male rabbits (E♂) as compared to the control group (C♂) (*P* < 0.05). However, significant differences between the two groups of females were not found. Except for the minimum diameter of the secondary osteons in females from the group E♀, patulin exposure did not influence sizes of Haversian canals or secondary osteons in any of the groups.

**Table 2:** Data of the primary osteons’ vascular canals, Haversian canals and secondary osteons in adult male rabbits intramuscularly exposed to patulin at dose 10 μg/kg body weight two times per week for 28 days (E♂) and control rabbits (C♂)

Measured structure	Group	N	Area (μm^2^)	Perimeter (μm)	Max. diameter (μm)	Min. diameter (μm)
Primary osteons’ vascular canals	C♂	124	320.76 ± 48.60	64.23 ± 4.87	11.13 ± 1.09	9.22 ± 1.02
	E♂	125	262.05 ± 58.01	58.01 ± 6.32	10.03 ± 1.43	8.34 ± 1.12
	*P* value		<0.05	<0.05	<0.05	<0.05
Haversian canals	C♂	85	388.51 ± 117.66	70.22 ± 10.84	12.14 ± 2.16	10.07 ± 1.69
	E♂	90	398.57 ± 130.97	70.87 ± 11.58	12.23 ± 2.18	10.21 ± 1.82
	*P* value		NS	NS	NS	NS
Secondary osteons	C♂	85	8846.06 ± 3950.00	332.25 ± 84.17	58.98 ± 16.79	45.46 ± 11.05
	E♂	90	9627.27 ± 4356.66	345.41 ± 77.95	60.44 ± 14.31	48.54 ± 11.71
	*P* value		NS	NS	NS	NS

*N* number of measured structures, *NS* non-significant differences

**Table 3:** Data of the primary osteons’ vascular canals, Haversian canals and secondary osteons in adult female rabbits intramuscularly exposed to patulin at dose 10 μg/kg body weight two times per week for 28 days (E♀) and the control rabbits (C♀)

Measured structure	Group	N	Area (μm^2^)	Perimeter (μm)	Max. diameter (μm)	Min. diameter (μm)
Primary osteons’ vascular canals	C*♀*	163	290.11 ± 85.10	61.75 ± 9.68	11.06 ± 2.27	8.33 ± 1.35
	E*♀*	163	281.05 ± 71.06	61.10 ± 9.50	10.87 ± 2.45	8.30 ± 1.21
	*P* value		NS	NS	NS	NS
Haversian canals	C*♀*	120	410.43 ± 110.65	72.35 ± 9.97	12.55 ± 2.04	10.33 ± 1.56
	E*♀*	107	411.23 ± 106.38	72.25 ± 9.73	12.49 ± 2.01	10.39 ± 1.36
	*P* value		NS	NS	NS	NS
Secondary osteons	C*♀*	120	7780.39 ± 2720.53	313.95 ± 52.25	54.79 ± 9.47	44.23 ± 9.03
	E*♀*	107	7495.80 ± 2585.41	311.99 ± 56.59	56.71 ± 11.36	41.04 ± 7.86
	*P* value		NS	NS	NS	P < 0.05

*N* number of measured structures, *NS* non-significant differences

## Discussion

Many experimental studies have confirmed toxic effects of patulin on humans and animals [2, 3, 8, 14, 22, 24]. Therefore, its presence in food is undesirable and should be monitored and controlled [37]. The results of our qualitative histological analysis correspond with those previously reported in rabbits [38–41]. The basic structural pattern of compact bone in the control rabbits was primary vascular longitudinal. In addition, primary vascular radial and/or dense Haversian and/or irregular Haversian bone tissues were present.

Patulin exposure caused marked alterations in bone microstructure in both sexes (E♂, E♀), although differences between the sexes were observed as well.

It is known that males and females vary in response to xenobiotic treatment and occupational exposures as a consequence of differences in body weight, length, body surface area, total body water content and kinetics and dynamics of xenobiotics [42]. Regarding bone morphology, males have greater bone size (resulting from bone acquired at the periosteal surface) and bone strength than females [43].

The skeletal sexual dimorphism is, according to Callewaert et al. [44], generally attributed to opposing sex steroid actions in males and females. Indeed, androgens stimulate periosteal bone formation whereas estrogens suppress it [45]. With respect to patulin toxicity, there is growing evidence that mycotoxins such as patulin may disturb the function of the endocrine system. Experimental studies have demonstrated significantly increased estradiol production by H295R cells following exposure to 5000 ng patulin per ml medium [46], and considerable higher levels of testosterone in growing male rats after gavage administration of 0.1 mg/kg bw/day for 60 or 90 days [47]. On the basis of these findings we hypothesize that the observed adverse effects of patulin on bone microstructure may be due to changed impact of sex hormones on bone turnover.

Generally, bone can adapt its composition and structure to changes in load [48]. This remarkable feature of bone is achieved by adaptive modeling and remodeling of the periosteal and endocortical surfaces of compact bone [49]. In males injected with patulin, the absence of subendosteal primary vascular longitudinal bone tissue may be due to intensive endosteal bone resorption caused by patulin toxicity. According to Szulc et al. [50], bone loss from the endocortical surface (contributing to bone fragility) may be compensated by deposition of new bone tissue on the periosteal surface as an adaptive response to maintain resistance to bending. We assume that the intensity of this process was enhanced also by patulin-stimulated production of testosterone. As a result of this adaptive mechanism, primary vascular longitudinal bone tissue (extending from the periosteal surface) into the middle area of *substantia compacta* was also present in rabbits from group E♂. On the other hand, apposition of bone tissue on the periosteal surface was not found in females given patulin. Periosteal apposition could probably be suppressed by a high level of estrogens. The changes in bone microstructure in group E♀ rabbits may also be due to adaptive responses of bone to patulin exposure. In contrast to inhibitory effects on periosteal expansion, estrogens have been shown to stimulate endosteal bone apposition in females [44]. This finding was also substantiated in our study. Female and male rabbits from the experimental groups had primary vascular longitudinal bone tissue in some areas of the middle part of compact bone. However, this type of bone tissue was expanded from periosteal surfaces in males exposed to patulin while it was expanded from endosteal surfaces in females. Moreover, due to vascular canal expansion from the endosteal borders, secondary osteons were marginalized within the periosteum and their number in the middle area of the bone was reduced.

The thickness of cortical bone is, generally, an important parameter in the evaluation of cortical bone quality. Since thickness measurement is a good predictor of skeletal mineralization, measurements of the cortical thickness of femoral shaft have been used extensively to estimate osteoporotic changes in the bone [51, 52]. The femoral cortical thickness was similar for both sexes of control rabbits. Indeed, males and females have (despite of some differences in bone microstructure) similar thickness of cortical bones [43, 53]. The increased cortical bone thickness in rabbits from both experimental groups could be associated with enhanced formation of bone tissue induced by sex hormones within the periosteum (in males) and the endosteum (in females) as a result of compensative mechanisms against patulin toxicity. It is known that estrogens play an important role in bone metabolism not only in females but also in males. However, males are more responsive to the stimulatory effects of androgens and less responsive to the inhibitory effects of estrogens. Therefore, they exhibit more pronounced periosteal enlargement [43]. Our results show that the impact of patulin on cortical bone thickness is sex-dependent in adult rabbits. Significant influence of patulin exposure on the size of the primary osteons’ vascular canals in male rabbits produces reduced bone vascularization. Intact primary vascular canal contains blood vessels [54] which are critical targets of toxic action of various xenobiotics. Taking this into account, the decreased size of the primary osteons’ vascular canals could be associated with vasoconstriction of blood vessels due to effects of patulin. This hypothesis is supported by Broom et al. [55] who reported transient vasoconstriction in rabbits, albeit their rabbits were exposed to patulin at a higher dose (0.08 mg/kg bw) than we used.

Absence of changes in histomorphometry of primary osteons’ vascular canals of group E♀ rabbits suggests sex-dependent effects of patulin on these structures. This sexual dimorphism can be again associated with the different influence of patulin-modified levels of sex hormones on vascular reactivity. Indeed, animal studies provide strong evidence that estradiol is an antihypertensive sex hormone, whereas testosterone is pro-hypertensive agent [56, 57].

Since patulin can enter the food chain, its levels mainly in apples and apple juices should be strictly monitored and controlled in order to minimize any potential risks to human health. Generally, experimental animals are unavoidable and indispensable research tools in the fields of bone toxicology. Therefore, our findings could be considered in assessing the health risks in experimental animals, as well as in humans following exposure to patulin.

## Conclusions

Intramuscular administration of patulin at dose 10 μg/kg bw twice per week for 28 days significantly affected cortical bone thickness in adult rabbits. The effects were sex-dependent. In males, absence of subendosteal primary vascular longitudinal bone and decreased size of the primary osteons’ vascular canals were found. In females, a lower number of secondary osteons (*P* < 0.05) in the middle area of the *substantia compacta* and occurrence of osteons near the periosteum were present. The results can be applied in experimental studies focusing on sex-related toxicity of xenobiotics on skeletal structure.

## References

[1] Fernándéz M, Simonían A, Berrada H, Ruiz MJ (2006). *In vitro* cytotoxicity of patulin, deoxynivalenol, nivalenol and zearalenone on CHO-K1 cells. Toxicol Lett.

[2] Reddy KRN, Salleh B, Saad B, Abbas HK, Abel CA, Shier WT (2010). An overview of mycotoxin contamination in foods and its implications for human health. Toxin Rev.

[3] Hussein SH, Brasel JM (2001). Toxicity, metabolism, and impact of mycotoxins on humans and animals. Toxicology.

[4] Bennett JW, Klich M (2003). Mycotoxins. Clin Microbiol Rev.

[5] Shephard GS (2008). Determination of mycotoxins in human foods. Chem Soc Rev.

[6] Alves I, Oliveira NG, Laires A, Rodrigues AS, Rueff J (2000). Induction of micronuclei and chromosomal aberrations by the mycotoxin patulin in mammalian cells: role of ascorbic acid as a modulator of patulin clastogenicity. Mutagenesis.

[7] Gonzáles-Osnaya L, Soriano JM, Moltó JC, Mañes J (2007). Exposure to patulin from consumption of apple-based products. Food Addit Contamin.

[8] Mahfoud R, Maresca M, Garmy N, Fantini J (2002). The mycotoxin patulin alters the barrier function of the intestinal epithelium: mechanism of action of the toxin and protective effects of glutathione. Toxicol Appl Pharmacol.

[9] Moake MM, Padilla-Zakour OI, Worobo RW (2008). Comprehensive review of patulin control methods in foods. Compr Rev Food Sci F.

[10] Neri F, Donati I, Veronesi F, Mazzoni D, Mari M (2010). Evaluation of *Penicillium expansum* isolates for aggressiveness, growth and patulin accumulation in usual and less common fruit hosts. Int J Food Microbiol.

[11] Burda K (1992). Incidence of patulin in apple, pear, and mixed fruit products marketed in New South Wales. J Food Prot.

[12] Forouzan Sh, Madadlou A (2014). Incidence of patulin in apple juices produced in West Azerbayjan Province, Iran. J Agr Sci Tech.

[13] Shepard GS, Leggott NL (2000). Chromatographic determination of the mycotoxin patulin in fruit and fruit juices. J Chromatogr A.

[14] Baert K, Devlieghere F, Amiri A, de Meulenaer B (2012). Evaluation of strategies for reducing patulin contamination of apple juice using a farm to fork risk assessment model. Int J Food Microbiol.

[15] Schumacher DM, Metzler M, Lehmann L (2005). Mutagenicity of the mycotoxin patulin in cultured Chinese hamster V79 cells, and its modulation by intracellular glutathione. Arch Toxicol.

[16] Glaser N, Stopper H (2012). Patulin: mechanism of genotoxicity. Food Chem Toxicol.

[17] Liu BH, Yu FY, Wu TS, Li SY, Su MC, Wang MC, Shih SM (2003). Evaluation of genotoxic risk and oxidative DNA damage in mammalian cells exposed to mycotoxins, patulin and citrinin. Toxicol Appl Pharmacol.

[18] Sharma RP (1993). Immuntoxicity of mycotoxins. J Dairy Sci.

[19] Dickens F, Jones HEH (1961). Carcinogenic activity of a series of reactive lactones and related substances. Br J Cancer.

[20] Wu T-S, Liao Y-C, Yu F-Y, Chang C-H, Liu B-H (2008). Mechanism of patulin induced apoptosis in human leukemia cells (HL-60). Toxicol Lett.

[21] Heussner AH, Dietrich DR, O’Brien E (2006). *In vitro* investigation of individual and combined cytotoxic effects of ochratoxin A and other selected mycotoxins on renal cells. Toxicol In Vitro.

[22] Puel O, Galtier P, Oswald IP (2010). Biosynthesis and toxicological effects of patulin. Toxins.

[23] Iwahashi Y, Hosoda H, Park JH, Lee JH, Suzuki Y, Kitagawa E, Murata SM, Jwa NS, Gu MB, Iwahashi H (2006). Mechanisms of patulin toxicity under conditions that inhibit yeast growth. J Agric Food Chem.

[24] Speijers GJ, Franken MA, van Leeuwen FX (1998). Subacute toxicity study of patulin in the rat: effects on the kidney and the gastro-intestinal tract. Food Chem Toxicol.

[25] Selmanoglu G (2006). Evaluation of the reproductive toxicity of patulin in growing male rats. Food Chem Toxicol.

[26] Kockaya EA, Selmanoglu G, Ozsoy N, Gül N (2009). Evaluation of patulin toxicity in the thymus of growing male rats. Arh Hig Rada Toksikol.

[27] Huff WE, Doerr JA, Hamilton PB, Hamann DD, Peterson RE, Ciegler A (1980). Evaluation of bone strength during aflatoxicosis and ochratoxicosis. Appl Microbiol.

[28] Maurice DV, Bodine AB, Rehrer NJ (1983). Metabolic effects of low aflatoxin B1 on broiler chickens. Appl Environ Microbiol.

[29] Neyt JG, Buckwalter JA, Carroll NC (1998). Use of animal models in musculoskeletal research. Iowa Orthop J.

[30] Gilsanz V, Roe TF, Gibbens DT, Schulz EE, Carlson ME, Gonzalez O, Boechat MI (1988). Effect of sex steroids on peak bone density of growing rabbits. Am J Physiol.

[31] Pearce AI, Richards RG, Milz S, Schneider E, Pearce SG (2007). Animal models for implant biomaterial research in bone: a review. Eur Cell Mater.

[32] Food and Agriculture Organization (FAO): Worldwide Regulations for Mycotoxins in Food and Feed in 2003 (2003). FAO food and nutrition papers 81.

[33] Martiniaková M, Omelka R, Grosskopf B, Sirotkin AV, Chrenek P (2008). Sex-related variation in compact bone microstructure of the femoral diaphysis in juvenile rabbits. Acta Vet Scand.

[34] Martiniaková M, Omelka R, Jančová A, Stawarz R, Formicki G (2010). Heavy metal content in the femora of yellow-necked mouse (*Apodemus flavicollis*) and wood mouse (*Apodemus sylvaticus*) from different types of polluted environment in Slovakia. Environ Monit Assess.

[35] Enlow DH, Brown SO (1956). A comparative histological study of fossil and recent bone tissues. Part I. Texas J Sci.

[36] de Ricqlés AJ, Meunier FJ, Castanet J, Francillon-Vieillot H, Hall BK (1991). Comparative microstructure of bone. Bone 3, bone matrix and bone specific products.

[37] Lawley R, Curtis R, Davis J (2012). The food safety hazard guidebook.

[38] Enlow DH, Brown SO (1958). A comparative histological study of fossil and recent bone tissues. Part III. Texas J Sci.

[39] Martiniaková M, Vondráková M, Fabiš M (2003). Investigation of the microscopic structure of rabbit compact bone tissue. Scripta medica (Brno).

[40] Martiniaková M, Omelka R, Chrenek P, Vondráková M, Bauerová M (2005). Age-related changes in histological structure of the femur in juvenile and adult rabbits: a pilot study. Bull Vet Inst Pulawy.

[41] Martiniaková M, Omelka R, Grosskopf B, Chovancová H, Massányi P, Chrenek P (2009). Effects of dietary supplementation of nickel and nickel-zinc on femoral bone structure in rabbits. Acta Vet Scand.

[42] Soldin OP, Chung SH, Mattison DR (2011). Sex differences in drug disposition. J Biomed Biotech.

[43] Rabijewski M, Papierska L, Dubey RK (2012). Osteoporosis in men—a crucial role of sex hormones. sex hormones.

[44] Callewaert F, Sinnesael M, Gielen E, Boonen S, Vanderschueren D (2010). Skeletal sexual dimorphism: relative contribution of sex steroids, GH-IGF1, and mechanical loading. J Endocrinol.

[45] Turner CH (2003). Periosteal apposition and fracture risk. J Musculoskeletal Neuron Interact.

[46] Frizzel C, Elliott CT, Connolly L (2014). Effects of the mycotoxin patulin at the level of nuclear receptor transcriptional activity and steroidogenesis *in vitro*. Toxicol Lett.

[47] Selmanoglu G, Kockaya EA (2004). Investigation of the effects of patulin on thyroid and testis, and hormone levels in growing male rats. Food Chem Toxicol.

[48] Seeman E (2007). The periosteum—a surface for all seasons. Osteoporos Int.

[49] Seeman E (2008). Bone quality: the material and structural basis of bone strength. J Bone Miner Metab.

[50] Szulc P, Seeman E, Duboeuf F, Sornay-Rendu E, Delmas PD (2006). Bone fragility: failure of periosteal apposition to compensate for increased endocortical resorption in postmenopausal women. J Bone Miner Res.

[51] Virtama P, Telkkae A (1962). Cortical thickness as an estimate of mineral content of human humerus and femur. Br J Radiol.

[52] Bloom RA, Bloom MB (1980). A comparative estimation of the combined cortical thickness of various bone sites. Skelet Radiol.

[53] Orwoll ES, Bilezikian JP, Vanderschueren D (2009). Osteoporosis in men: the effects of gender on skeletal health.

[54] Greenlee DM, Dunnell RC (2010). Identification of fragmentary bone from the Pacific. J Archaeol Sci.

[55] Broom WA, Bülbring E, Chapman CJ, Hampton JWF, Thomson AM, Ungar J, Wien R, Woolfe G (1944). The pharmacology of patulin. Br J Exp Pathol.

[56] Dubey RK, Oparil S, Imthurn B, Jackson EK (2002). Sex hormones and hypertension. Cardiovasc Res.

[57] Hutchison SJ, Sudhir K, Chou TM, Sievers RE, Zhu BQ, Sun YP, Deedwania PC, Glantz SA, Parmley WW, Chatterjee K (1997). Testosterone worsens endothelial dysfunction associated with hypercholesterolemia and environmental tobacco smoke exposure in male rabbit aorta. J Am Coll Cardiol.

